# A Cross-Sectional Study of the Knowledge, Attitude, and Practice of Self-Medication Among the General Population in the Western Region of Saudi Arabia

**DOI:** 10.7759/cureus.29944

**Published:** 2022-10-05

**Authors:** Mohammed E Almalki, Fahad S Almuqati, Muhannad O Alwezainani, Saleh Y Makki, Majed A Alqasem, Faisal F Alsharif, Abdurahman Hassan-Hussein

**Affiliations:** 1 Medicine, Umm Al-Qura University, Mecca, SAU; 2 Community Medicine, Umm Al-Qura University, Mecca, SAU

**Keywords:** healthcare-seeking behavior, over the counter, public health, self-medication, drug

## Abstract

Introduction

Self-medication (SM) is defined as consuming pharmaceutical drugs without the advice of a physician for either diagnosis or treatment. Reliance on self-medication has become a more common worldwide issue and now plays a major role in self-care. However, the practice is linked to many risks for patients and the whole community. This study assesses knowledge, attitudes, and practices associated with self-medication in the western region of Saudi Arabia.

Methods

This is an observational questionnaire-based cross-sectional study conducted over two months, between January and March 2022. The survey comprised 29 questions adapted from similar studies and was translated into Arabic to fit the study population. All residents of the three major cities, Makkah, Jeddah, and Taif, were included; the population under 18 years of age and health workers were excluded. We used OpenEpi version 3.0 (www.OpenEpi.com) for sample size calculation and Statistical Package for Social Sciences (IBM Corp, Armonk, USA) was used for data analysis.

Results

Most of the participants (67.7%) declared that they practiced self-medication: (28.6%) men and (39.1%) women. Self-medicating for different indications showed differences between men and women but without statistical significance. Major indications for self-medicating were headache (45.3%), cough, cold/flu (42.7%), and fever (34.0%). The primary reasons participants gave for choosing to self-medicate were easy availability of the medicines (41.4%) and that they were treating a minor illness (40.8%). Many types of medicines were used, most commonly analgesics (44.0%) and antipyretics (43.6%).

Conclusion

The practice of self-medication is high among the population in Makkah, Jeddah, and Taif. Educating the public on the consequences and adverse effects is necessary.

## Introduction

Self-medication, buying and taking medications without a physician’s advice or supervision, is a common practice worldwide nowadays, and a part of one’s self-care [1,2]. When done responsibly [3], self-medicating can be beneficial, allowing individuals to take responsibility for their health and make decisions in treating short-lived, mild illnesses. This, in fact, is encouraged by drug policies in many countries, as it cuts costs for governments and lessens the burden on health care providers, enabling them to direct their attention to more serious illnesses [4].

The prevalence of self-medication varies widely between countries. In a review of 140 studies covering a total of 189,278 individuals of all ages, prevalence rates ranged from as little as 0.1% to 100%, and many studies revealed high rates, for example, 65% in Finland, 78% in Yemen, Saudi Arabia (SA), and Uzbekistan, and 88-92% in Thailand [5]. A study from Egypt found a prevalence of 96%, showing an increase among all socioeconomic levels from the results of previous studies in the country [6]. The commonest medicines used without a prescription include antibiotics 59%, non-steroidal anti-inflammatory drugs 43.31%, and cough and cold medicines 13.9% [5].

The magnitude and patterns of nonprescription drug use should be investigated for different types of medicines in different regions, especially in view of the possible associated health risks. For example, the WHO has cautioned against growing antibiotic resistance due to self-medication with antibiotics [7]. In Makkah during the Hajj season of 2015, the prevalence of non-prescription antibiotic use among pilgrims was over 87% [8]. And among 1140 families in Egypt, over half 53.9% used antibiotics without a prescription [9]. Other possible risks associated with self-medication include incorrect self-diagnosis, masking of an underlying severe disease, and subsequent failure to seek medical advice promptly. More severe risks are failure to recognize contraindications and potential interactions with food or other drugs, mistakes in the method of administration, dosage, storage, and choice of medicine, and the risk of drug dependence or abuse. At the community level, improper self-medication produces an increment in drug-induced disease with an increase in public health expenditure [10]. Previous reports from SA show that the practice of buying and selling non-prescription medications is common [11,12]. In a study from Saudi Arabia (SA), 81.3% of participants practiced self-medication; among them, 11.47% experienced complications and harmful side effects [13]. Additionally, irrational self-medication practices may increase the incidence of misdiagnosis and drug resistance [14,15]. Furthermore, according to a study conducted in Karachi, Pakistan, the misuse of nonprescription drugs has become a serious problem among adolescents; the high involvement of the current generation with social media has increased their exposure to pharmaceutical advertisements, which motivates them toward self-medication [16].

As data on self-medication in the Western region of SA are insufficient, this study assesses knowledge, attitudes, and practices among the general population of that region related to self-medication, aiming to determine its prevalence and identify factors and sources of information influencing and contributing to its practice.

## Materials and methods

This observational cross-sectional study was conducted by a self-administered online survey over two months between January and March 2022. The study targeted the population of Makkah, Jeddah, and Taif. All residents of the three cities were included, while health care workers and the population under 18 years old were excluded. Participants were selected using a non-sampling convenience sampling technique, and data were collected online using Google Forms. Based on a review of similar studies the research team developed a 27-item questionnaire divided into two sections. The first section (seven questions) focused on sociodemographic characteristics (sex, age group, nationality, educational level, monthly income, chronic diseases). The second section (20 questions) assessed the participants' knowledge, attitude, and practice of self-medication. The questionnaire was adapted from similar studies [17,18] and translated into Arabic to suit the study population after consultation with an epidemiologist and a family medicine physician. We used OpenEpi (Version 3.0, www.OpenEpi.com) for sample size calculation: a minimum sample size of 385 was required for the study, considering a 95% confidence interval (CI) and anticipated frequency of 50%, and design effects of 1. Consent to participate was taken from participants electronically.

Data analysis

We included both qualitative and quantitative data and used IBM SPSS Statistics Version 25.0 (IBM Corp., Armonk, USA) for data analysis. Descriptive statistics were used for proportions. Associations between demographic characteristics and understanding perceptions and practices were tested using the Chi-square test. P-values were calculated using the Chi-square test, and an alpha level of 0.05 or less was considered significant. Microsoft Excel (Microsoft Corporation, Redmond, USA) was used for data analysis and preparing charts, graphs, and diagrams.

Ethical considerations

This study was approved by the Medical Research Ethics Committee at Umm Al-Qura University (No. HAPO-02-k-012-2021-12-868). Consent to participate was taken from participants electronically.

## Results

A total of 647 residents participated in the survey: women constituted 56.6% (Table 1). Almost two-thirds of the participants were under 40 years old: 33.8% were aged between 18-28, and 28.0% were between 29-39. A greater proportion of participants were from Makkah (39.3%), and the majority were Saudi citizens (72.5%). Most participants were university or high school graduates - 51.2% and 28.6%, respectively. Two-thirds had no chronic illness - 63.6%.

**Table 1:** Demographic characteristics No: Number

Characteristics		No (%)
Sex	Female	366 (56.6%)
	Male	281 (43.4%)
Age group	18 – 28	219 (33.8%)
	29 – 39	181 (28.0%)
	40 – 50	147 (22.7%)
	Over 50	100 (15.5%)
City	Makkah	254 (39.3%)
	Jeddah	202 (31.2%)
	Taif	191 (29.5%)
Nationality	Saudi	469 (72.5%)
	Non-Saudi	178 (27.5%)
Educational level	No formal education	35 (5.4%)
	Elementary school	45 (7.0%)
	Intermediate school	51 (7.9%)
	High school	185 (28.6%)
	University graduate	331 (51.2%)
Monthly income (Saudi Riyals)	less than 1000	112 (17.3%)
	1000 – 3000	104 (16.1%)
	3001 – 7000	172 (26.6%)
	7001 – 10,000	169 (26.1%)
	More than 10,000	90 (13.9%)
Chronic diseases	Yes	235 (36.3%)
	No	412 (63.7%)

Most participants (67.7%) declared that they practiced self-medication, 28.6% men and 39.1% women, with no significant difference (P > 0.05) (Table 2).

**Table 2:** Practice of self-medication N: Number

	Response	Male		Female		Total (%)	P Value
Do you practice self-medication?		N	%	N	%		
	Yes	185	28.6%	253	39.1%	438 (67.7%)	0.423
	No	96	14.8%	113	17.5%	209 (32.3%)	

Table 3 shows that self-medication for different medical problems differed between the men and women but with no statistical significance (P > 0.05). Common indications for self-medicating included headache 45.3%, cough, cold/flu 42.7%, and fever 34.0%. While ear problems were the least (12.8%).

**Table 3:** Reasons for self-medicating N: Number

Item	Male		Female		Total (%)	P Value
Indication for self-medicating	N	%	N	%		
Headache	121	18.7	172	26.6	293 (45.3%)	0.359
Cough, cold/Flu	114	17.6	162	25.0	276 (42.7%)	0.389
Fever	91	14.1	129	19.9	220 (34.0%)	0.498
Stomachache	77	11.9	132	20.4	209 (32.3%)	0.024
Diarrhea	75	11.6	118	18.2	193 (29.8%)	0.149
Menstrual symptoms	0	0	165	25.5	165 (25.5%)	2.412
Rash/allergies	35	5.4	62	9.6	97 (15.0%)	0.142
Anxiety/depression	49	7.6	67	10.4	116 (17.9%)	0.856
Ear problems	36	5.6	47	7.3	83 (12.8%)	1.000
Vomiting	41	6.3	71	11.0	112 (17.3%)	0.134
Eye infections	42	6.5	57	8.8	99 (15.3%)	0.913
Skin problems	38	5.9	65	10.0	103 (15.9%)	0.177
Toothache	69	10.7	100	15.5	169 (26.1%)	0.481
Insomnia	69	10.7	88	13.6	157 (24.3%)	0.954
Pain	62	9.6	71	11.0%	133 (20.6%)	0.463
Reason for self-medicating						
Minor illness	112	17.3	152	23.5	264 (40.8%)	0.728
Sufficient pharmacological knowledge	95	14.7	143	22.1	238 (36.8%)	0.196
Quick relief	97	15.0	122	18.9	219 (33.8%)	0.816
Lack of time to consult doctor	97	15.0	149	23.0	246 (38.0%)	0.127
Cost effectiveness	107	16.5	140	21.6	247 (38.2%)	1.000
Easy availability of medicine	115	17.8	153	23.6	268 (41.4%)	0.885
Emergency use	79	12.2	119	18.4	198 (30.6%)	0.264
Type of self-prescribed medicine						
Analgesics	121	18.7	164	25.3	285 (44.0%)	0.716
Antipyretics	121	18.7	161	24.9	282 (43.6%)	0.876
Antidiarrheals	55	8.5	91	14.1	146 (22.6%)	0.133
Antiemetics	57	8.8	92	14.2	149 (23.0%)	0.174
Antibiotics	75	11.6	91	14.1	166 (25.7%)	0.662
Antacids	61	9.4	97	15.0	158 (24.4%)	0.189
Sedatives	62	9.6	95	14.7	157 (24.3%)	0.293
Anti-allergic	66	10.2	86	13.3	152 (23.5%)	1.000
Vitamins	99	15.3	152	23.5	251 (38.8%)	0.122
Ophthalmic preparations	49	7.6	74	11.4	123 (19.0%)	0.428
Cosmetic products	41	6.3	68	10.5	109 (16.8%)	0.216

The main reasons for choosing to self-medicate were that the medicines were easily available (41.4%) and the illnesses treated were minor (40.8%). Other reasons included cost-effectiveness (38.2%), not having time to consult a doctor (38.0%), and having sufficient knowledge of the medicines (36.8%).

As shown in Table 3, various types of medicines were used without prescription, including analgesics 44.0%, antipyretics 43.6%, vitamins 38.8%, and antibiotics 25.7%. Cosmetic products were the least used (16.8%).

Table 4 represents participants’ opinions of the factors that influenced them toward self-medicating. The most common factors were having a previous experience with the medicine (60.6%) and having a previous doctor’s prescription for the medicine (52.6%), while 33.7% were influenced by the opinion of a family member. Advertisements had the least influence (17.9%).

**Table 4:** Factors influencing the practice of self-medication N: Number

Factor	Male		Female		Total (%)	P Value
	N	%	N	%		
Family member’s opinion	91	14.1	127	19.6	218 (33.7%)	0.594
Friend’s opinion	80	12.4	94	14.5	174 (26.9%)	0.482
Recommendation from locals	97	15.0	103	15.9	200 (30.9%)	0.098
Previous prescription from a doctor	147	22.7	193	29.8	340 (52.6%)	0.979
Own experience	162	25.0	230	35.5	392 (60.6%)	0.208
Advertisement	43	6.6	73	11.3	116 (17.9%)	0.155

We assessed the participants’ knowledge related to self-medication (Table 5). Most participants who used self-prescribed medicines for different illnesses checked the expiry date before taking the medicine 61.7%, and 58.9% tended to check the information insert. An equal percentage of participants (49.3%) had previously heard about self-medication and knew that they should complete the dose when self-medicating. However, there was a statistically significant difference between the number of men and women who had previously heard of self-medication (P < 0.05).

**Table 5:** Participant's knowledge of self-medication N: Number

	Male		Female		Total (%)	P Value
	N	%	N	%		
Previously heard of self-medication	125	19.3	194	30.0	319 (49.3%)	0.038
Knows about completing duration of treatment	137	21.2	182	28.1	319 (49.3%)	0.868
Checks information insert	154	23.8	227	35.1	381 (58.9%)	0.077
Checks expiration date before use	167	25.8	232	35.9	399 (61.7%)	0.345

As shown in Table 6, a statistically significant number of participants agreed that they would advise family and friends to self-medicate, had an idea about drugs that have side effects, and believed that mild medical problems do not need medication and that medicines should not be sold without a prescription.

**Table 6:** Attitudes related to self-medication

	City	Yes	No	P value
Self-medication is part of self-care	Makkah	143	111	2.375
	Jeddah	170	32	
	Taif	95	96	
Would you advise family and friends to self-medicate?	Makkah	118	136	0.002
	Jeddah	125	77	
	Taif	92	99	
Do you have an idea about drugs that have side effects?	Makkah	140	114	0.036
	Jeddah	111	91	
	Taif	84	107	
Are you concerned that increasing the drug dose can be hazardous to health?	Makkah	169	85	1.168
	Jeddah	155	47	
	Taif	95	96	
In case of side effects, a physician’s help is needed	Makkah	170	84	3.414
	Jeddah	176	26	
	Taif	96	95	
Mild medical problems do not need medication	Makkah	142	112	0.000
	Jeddah	142	60	
	Taif	94	97	
We do not need physicians	Makkah	94	160	2.077
	Jeddah	20	182	
	Taif	59	132	
Taking medicine without enough knowledge can be harmful	Makkah	167	87	3.206
	Jeddah	182	20	
	Taif	98	93	
Do you agree that medicines should not be sold without a prescription?	Makkah	143	111	0.003
	Jeddah	85	117	
	Taif	84	107	

**Figure 1:**
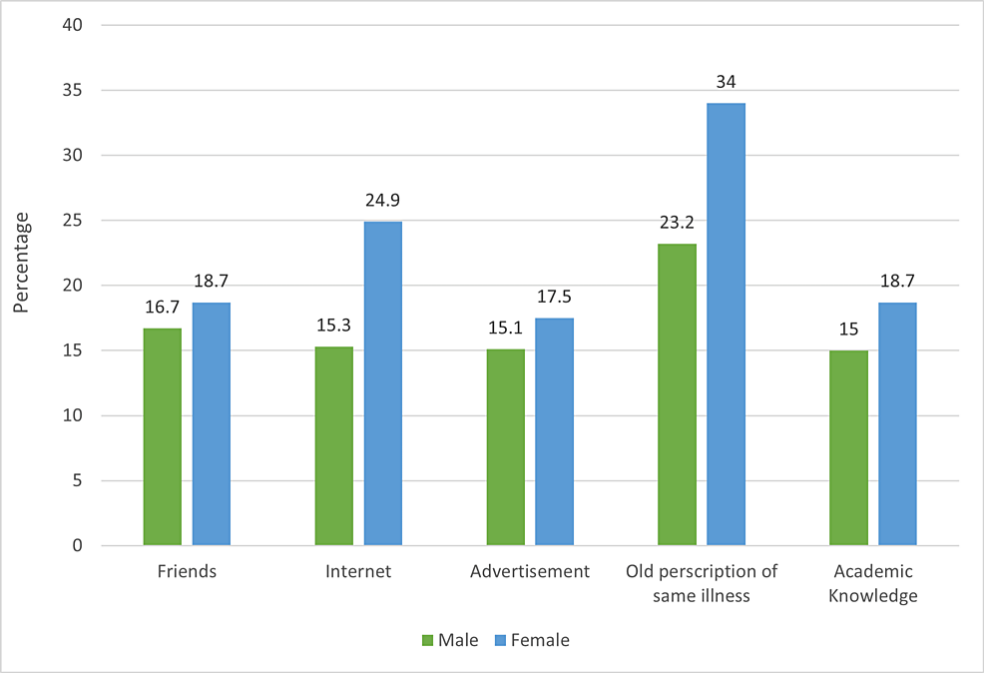
Source of information when self-medicating

Figure 1 represents the sources of information participants rely on for information when self-medicating. The most frequently chosen source among the men and women was an old prescription for a common illness (23.2% and 34.0%, respectively); the internet was the second most frequent.

**Figure 2:**
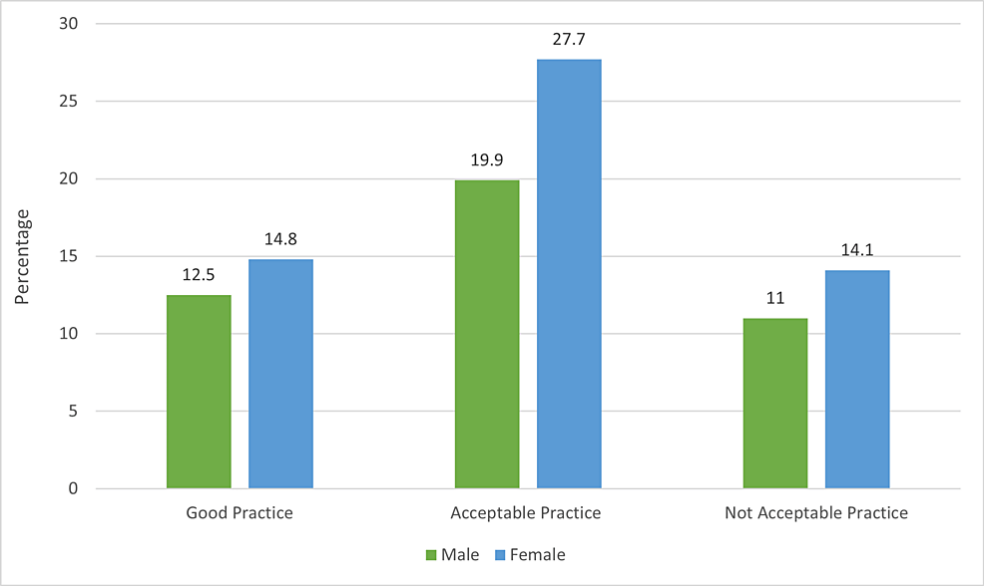
Participant attitude toward self-medication

Figure 2 represents the participants’ approach to self-prescribing medications for self-health care. The figure shows that the participants’ concept of self-medication was classified into three categories: good practice, acceptable practice, and unacceptable practice. Among them, 19.9% of the men and 27.7% of the women considered self-medication an acceptable practice.

## Discussion

Incidence

In the literature, the highest rates of self-medication have been reported in Eastern European and Asian countries [19]. In the present study, out of 647 survey respondents, 67.7% practiced self-medication. These findings align with previous studies in SA [20,21]. One of the most important factors leading to this phenomenon is the nonadherence of pharmacies to the country’s pharmacy law [20]. Our findings also support a study from Ethiopia that revealed women to be more prone to SM [22]. 

Reasons for self-medication

Among our participants, the most agreed-upon motive for self-medicating was the easy availability of medicines. This was expected since many types of medications are easily attainable and accessible in most households. Additionally, our survey showed that minor illnesses are among the leading reasons for self-medicating. For instance, a simple tension headache is considered a minor illness, and its treatment, paracetamol, is readily at hand. Another viable cause is cost-effectiveness, understandably, because purchasing medications from a local pharmacy is more affordable than seeking medical advice and paying consultation fees. Regarding indications for self-medicating, headache was the most prominent among the respondents. In Arab countries, headache is the most prevalent neurological symptom, although is underdiagnosed and undertreated [23]. Additionally, a meta-analysis from Iran demonstrated that fever, headache, and cough were the most notable triggers for self-medicating [24]. The second most frequent indication for self-medicating was cough and cold/flu. Most people are familiar with these symptoms, and because they commonly perceive that colds usually present with mild symptoms, they often tend to self-medicate rather than make the effort to book an appointment for a medical consultation. There is also a belief that only safe medicines are sold without a prescription and that over-the-counter medicines usually do not have side effects [25].

Type of medicine

The type of self-prescribed medicine is an imperative aspect in this study. One crucial concern is antimicrobial drug resistance. Despite all awareness campaigns by the Ministry of Health on antibiotic usage, antibiotics were used without prescription by one-fourth of the participants in this study and were the most frequent prescription medicine taken without a prescription in a study from the central region of SA [26]. This inappropriate use is of concern, as it may increase the probability of drug resistance [27]. Further, 80% of the respondents in another local study knew about the adverse effects of antibiotics, yet continued to take them [28]. Regulations and control over nongovernment pharmacies must therefore be in effect. We did not collect more information on the types of analgesics and frequency of use; however, such details would be imperative to assess how they are used. For instance, prolonged or excessive use of analgesics, such as nonsteroidal anti-inflammatory drugs, may have harmful effects. Such situations must be appropriately addressed to determine an adequate course of action. Vitamins were used as self-medication by 38.8% of the participants. There is a possibility that vitamins may be abused because many people might not consider them to be medicines [29].

Influencing factors

The most frequent factors that influenced participants to self-medicate were previous use of the medicine followed by having a doctor’s prescription from a previous ailment. This was consistent with a study in Ethiopia [30]. In fact, having previously used self-medication was nearly two times more likely to lead to self-medication than having no previous experience [31]. Additionally, participants in a study from France did not consider the use of medication from an old prescription to be self-medication [32].

Attitudes

Some individuals grow so confident in self-medicating that they attempt to give recommendations to close relatives, spreading erroneous information and the practice of self-medication among others. Adding to that, people often repeat certain medications based on former recommendations by their physician, especially if they experience similar symptoms; however, patients may misdiagnose themselves. This phenomenon is seen in countries of both low and high income [33]. Despite mild illness being the most common reason for self-medication, many participants in our study agreed that mild illnesses do not require medication.

Knowledge

Our results show that 49.3% of the respondents had heard about self-medication, in contrast to a study conducted in western Uganda, which reported a percentage of 97% [34]. Also, only half of our participants knew that they must complete the duration of treatment. This is consistent with several studies reporting inappropriate practices like obtaining an insufficient quantity of the medicine [35] and stopping medications once symptoms improve [36,37]. Additionally, about two-thirds of our participants checked the expiration date on their medicines, consistent with other similar reports [38].

Limitation

This study has a few limitations. First, recall bias may have influenced results. Second, the study included only one region in SA and, hence, cannot be generalized to the whole country. Also, the types of chronic diseases participants had were not specified. This was a cross-sectional study: variables were collected only once and, consequently, the data provided should be carefully interpreted before establishing a relationship.

## Conclusions

Our study found a high prevalence of self-medication practice among the population in Makkah, Jeddah, and Taif. However, the population's knowledge, attitude, and practice regarding self-medication were insufficient. Therefore, the practice should be considered a public health problem, as it may lead to medication misuse. We recommend that better awareness be adopted among people about the necessity of consulting a physician before taking any medicine and the importance of observing label instructions. It would also be helpful to use more explicit language in describing the dosage and frequency of use of prescribed drugs to improve comprehension, especially among patients with limited literacy.
