# Correction: A Cross-Sectional Study of the Knowledge, Attitude, and Practice of Self-Medication Among the General Population in the Western Region of Saudi Arabia

**DOI:** 10.7759/cureus.c90

**Published:** 2023-01-04

**Authors:** Mohammed E Almalki, Fahad S Almuqati, Muhannad O Alwezainani, Saleh Y Makki, Majed A Alqasem, Faisal F Alsharif, Abdurahman Hassan-Hussein

**Affiliations:** 1 Medicine, Umm Al-Qura University, Mecca, SAU; 2 Community Medicine, Umm Al-Qura University, Mecca, SAU

This article has been corrected at the request of the authors due to the incorrect spelling of the last name of the third author. "Muhannad O. Alotaibi" has been changed to "Muhannad O. Alwezainani". The authors deeply regret that this error was not corrected prior to submission and publication.

